# Timely Extracorporeal Cardiopulmonary Resuscitation for Intraoperative Cardiac Arrest Caused by Suspected Bone Cement Implantation Syndrome: A Case Report

**DOI:** 10.1002/ccr3.73439

**Published:** 2026-08-31

**Authors:** Yuta Kikuchi, Takashi Uchiyama, Hidehiko Nakamura

**Affiliations:** ^1^ Department of Cardiology Kashiwa Kosei General Hospital Kashiwa Japan; ^2^ Department of Cardiology Sanaikai General Hospital Misato Japan

**Keywords:** bone cement implantation syndrome, cardiac arrest, extracorporeal cardiopulmonary resuscitation, extracorporeal membrane oxygenation, perioperative cardiac arrest

## Abstract

Bone cement implantation syndrome should be suspected in patients with sudden circulatory collapse during cemented orthopedic surgery. Prompt recognition and timely extracorporeal cardiopulmonary resuscitation may improve neurological outcomes in carefully selected patients.

## 1 Introduction

Bone cement implantation syndrome (BCIS) is a potentially life‐threatening complication associated with cemented orthopedic procedures. It is characterized by sudden hypoxemia, hypotension, pulmonary hypertension, and, in severe cases, cardiovascular collapse or cardiac arrest. Although its exact pathophysiology remains incompletely understood, embolization of intramedullary contents and vasoactive effects of bone cement are considered major contributing mechanisms.

Cardiac arrest caused by BCIS is uncommon but carries an extremely poor prognosis. When conventional cardiopulmonary resuscitation fails, extracorporeal cardiopulmonary resuscitation (ECPR) using veno‐arterial extracorporeal membrane oxygenation (VA‐ECMO) may provide temporary circulatory support while the reversible pathophysiology recovers. However, the optimal timing and indications for ECPR in BCIS have not been established.

Here, we report a case of catastrophic perioperative cardiac arrest strongly suspected to be caused by BCIS, in which prompt clinical recognition of a potentially reversible etiology and timely initiation of extracorporeal cardiopulmonary resuscitation (ECPR) resulted in favorable neurological recovery. This case emphasizes the importance of early multidisciplinary decision‐making in patients with suspected BCIS.

## 2 Case History/Examination

An 86‐year‐old woman with chronic heart failure underwent cemented hemiarthroplasty for a femoral neck fracture. Her medical history included hypertension, chronic heart failure, persistent atrial fibrillation, previous cerebral infarction, rheumatoid arthritis, and dementia. Preoperative transthoracic echocardiography demonstrated a left ventricular ejection fraction of 37.5% with diffuse left ventricular hypokinesis and severe mitral regurgitation.

The procedure was performed under general anesthesia with continuous electrocardiography, noninvasive blood pressure monitoring, pulse oximetry, and end‐tidal carbon dioxide (EtCO_2_) monitoring. Before bone cement insertion, her blood pressure was 125/75 mmHg, heart rate was 75 beats/min, SpO_2_ was 98%, and EtCO_2_ was 25 mmHg. Approximately 3 min after bone cement insertion, her blood pressure decreased abruptly to 65/38 mmHg, heart rate to 50 beats/min, SpO_2_ to 85%, and EtCO_2_ to 10 mmHg. Arterial blood gas analysis obtained at that time showed a pH of 7.24, PaCO_2_ of 40 mmHg, PaO_2_ of 60 mmHg, and lactate level of 2.8 mmol/L. Intravenous ephedrine and phenylephrine were administered without a significant hemodynamic response. Rapid fluid infusion, continuous norepinephrine (0.2 μg/kg/min), continuous dobutamine (10 μg/kg/min), and intravenous adrenaline (50 μg) were subsequently administered; however, circulatory collapse progressed despite these interventions.

The orthopedic procedure was immediately abandoned, and the surgical wound was closed. Ten minutes after the initial hemodynamic collapse, the patient developed pulseless electrical activity (PEA). Cardiopulmonary resuscitation was initiated immediately, resulting in a no‐flow time of 0 min. Two cycles of intravenous adrenaline (1 mg) were administered. Because ROSC could not be achieved with conventional resuscitation, ECPR was considered. Given the witnessed in‐hospital cardiac arrest, absence of no‐flow time, suspected reversible etiology, and functional status sufficient to undergo surgery immediately before the event, ECPR was considered appropriate despite the patient's advanced age. The decision to proceed with ECPR was made by two cardiologists in consultation with the treating orthopedic surgeon. While cardiopulmonary resuscitation was continued, the patient was transferred to the catheterization laboratory.

## 3 Methods

Approximately 10 min after the onset of the first PEA arrest, ROSC was achieved shortly after arrival at the catheterization laboratory; however, profound hemodynamic instability persisted. Transthoracic echocardiography performed after the initial ROSC demonstrated right ventricular dilatation and hypokinesis, a D‐shaped left ventricle, and severe tricuspid regurgitation with a peak tricuspid regurgitation velocity of 4.0 m/s, suggesting marked right ventricular pressure overload. Coronary angiography revealed no significant coronary artery stenosis, and pulmonary angiography demonstrated no obvious filling defects.

Considering the close temporal relationship with bone cement insertion and the characteristic clinical findings, BCIS was considered the most likely diagnosis after exclusion of other major causes of circulatory collapse. During further evaluation, the patient developed recurrent PEA approximately 15 min after the initial ROSC. VA‐ECMO support was established 14 min after the recurrent arrest. Thus, the first and second episodes of conventional CPR lasted approximately 10 and 14 min, respectively, with an intervening period of spontaneous circulation of approximately 15 min. The cumulative low‐flow time was approximately 24 min. Because BCIS was considered the most likely diagnosis and a potentially reversible cause of cardiac arrest, VA‐ECMO was selected as ECPR. Return of spontaneous circulation was achieved shortly after initiation of VA‐ECMO, followed by progressive hemodynamic stabilization.

## 4 Conclusions and Results

The patient was successfully weaned from VA‐ECMO on postoperative day 1 and extubated on postoperative day 2. No clinically significant postoperative bleeding complications were observed despite perioperative anticoagulation for VA‐ECMO. Rehabilitation was initiated on the day of extubation, and the patient recovered the ability to walk. She had no apparent new neurological deficits and was transferred to a rehabilitation hospital on postoperative day 30. The clinical timeline of the patient's course is summarized in Table 1.

**TABLE 1:** Clinicaltime line of the patient's course from bone cement insertion to recovery.

Event	Details
Bone cement insertion	
↓ 3 min	
Hemodynamic collapse	BP 65/38 mmHg; HR 50 beats/min; SpO_2_ 85%; EtCO_2_ 10 mmHg
↓ 10 min	
Cardiac arrest	Pulseless electrical activity (PEA)
Cardiopulmonary resuscitation	CPR initiated immediately; no‐flow time 0 min; CPR continued during transfer to the catheterization laboratory
↓ 10 min	
Transient ROSC	Achieved shortly after arrival at the catheterization laboratory
Diagnostic evaluation	Echocardiography: RV dilatation and hypokinesis, D‐shaped LV, severe TR (TR Vmax 4.0 m/s); coronary angiography: no significant stenosis; pulmonary angiography: no obvious filling defects
↓ 15 min	
Recurrent PEA	
↓ 14 min	
VA‐ECMO initiation	ECPR
	Cumulative low‐flow time: approximately 24 min
↓	
Sustained ROSC	Progressive hemodynamic stabilization
Outcome	POD 1: VA‐ECMO weaning POD 2: Extubation and rehabilitation initiation POD 30: Transfer to rehabilitation hospital; ambulatory

## 5 Discussion

Bone cement implantation syndrome (BCIS) is a rare but potentially catastrophic complication of cemented orthopedic surgery. Although transient hypoxemia or hypotension is relatively common, progression to cardiac arrest is uncommon and is associated with a very high mortality rate. Therefore, rapid recognition and immediate intervention are essential [1].

The pathophysiology of BCIS is considered multifactorial. Increased intramedullary pressure during cement insertion may force fat, bone marrow, and other debris into the pulmonary circulation, resulting in acute pulmonary vascular obstruction and right ventricular overload [2, 3]. In addition, vasoactive substances released by bone cement may induce systemic vasodilation and further impair hemodynamics [1, 4]. In the present case, the abrupt onset of hypotension, hypoxemia, and decreased end‐tidal carbon dioxide immediately after bone cement insertion, together with echocardiographic evidence of acute right ventricular overload, supports BCIS as the most likely clinical diagnosis.

Several reports have described successful use of veno‐arterial extracorporeal membrane oxygenation (VA‐ECMO) for severe BCIS [5, 6, 7]. However, because such cases remain uncommon, the optimal indications and timing for extracorporeal cardiopulmonary resuscitation (ECPR) have not been established. The educational value of the present case lies in the prompt clinical recognition of suspected BCIS as a potentially reversible cause of catastrophic perioperative cardiac arrest and the timely decision to initiate ECPR before definitive confirmation of the diagnosis. This case emphasizes the importance of clinical judgment and multidisciplinary decision‐making when managing catastrophic perioperative circulatory collapse.

Current resuscitation guidelines suggest that ECPR may be considered as a rescue therapy for selected patients with refractory cardiac arrest when a potentially reversible cause is present and timely implementation is feasible [8]. Patient selection generally considers factors such as witnessed arrest, immediate high‐quality CPR, short no‐flow and low‐flow times, a potentially reversible etiology, and the absence of severe frailty or major comorbidities [8]. Advanced age is generally associated with a lower likelihood of favorable outcome and should be considered when selecting candidates for ECPR. In the present case, despite the patient's advanced age, the arrest was witnessed in hospital, the no‐flow time was zero, conventional CPR was initiated immediately, transient ROSC was achieved, and BCIS was considered a potentially reversible cause. These favorable features supported an individualized multidisciplinary decision to initiate ECPR. Nevertheless, this case should not be interpreted as supporting routine ECPR for elderly patients with BCIS; careful patient selection and the ability to establish extracorporeal support rapidly remain essential.

Another important point is that the diagnosis of BCIS is primarily clinical and remains a diagnosis of exclusion. No definitive diagnostic test exists for BCIS [1]. In the present case, coronary angiography showed no significant coronary artery stenosis, and pulmonary angiography showed no obvious filling defects, reducing the likelihood of acute coronary syndrome and massive pulmonary thromboembolism, respectively. However, pulmonary angiography cannot exclude microembolization, and fat or bone marrow emboli associated with BCIS may not be visualized on routine angiography [1, 2, 3]. Therefore, the diagnosis of BCIS in this case remained presumptive and was based on the close temporal relationship with bone cement insertion, the characteristic hemodynamic and respiratory deterioration, echocardiographic evidence of acute right ventricular overload, and the exclusion of other major causes of cardiac arrest. Delaying mechanical circulatory support until diagnostic certainty is achieved may reduce the likelihood of a favorable neurological outcome.

Risk factors for BCIS include advanced age, impaired cardiopulmonary function, and osteoporotic fractures [1, 9, 10]. Our patient had multiple predisposing factors, including chronic heart failure with reduced left ventricular systolic function and advanced age, which may have contributed to the severity of the event.

This case reinforces the importance of maintaining a high index of suspicion for BCIS in patients who develop sudden circulatory collapse during cemented orthopedic surgery. When conventional cardiopulmonary resuscitation fails, timely ECPR should be considered because the underlying pathophysiology may be reversible. Rather than advocating routine use of VA‐ECMO for all patients with BCIS, this report emphasizes that prompt recognition, multidisciplinary decision‐making, and timely ECPR may improve survival and neurological outcomes in carefully selected patients.

## Author Contributions

**Hidehiko Nakamura:** writing – review and editing. **Takashi Uchiyama:** supervision, writing – review and editing. **Yuta Kikuchi:** conceptualization, investigation, data curation, writing – original draft, writing – review and editing.

## Funding

The authors have nothing to report.

## Ethics Statement

This study was conducted in accordance with the Declaration of Helsinki. Written informed consent was obtained from the patient and the patient's family for publication of this case report.

## Conflicts of Interest

The authors declare no conflicts of interest.

## Data Availability

The data that support the findings of this study are available on request from the corresponding author. The data are not publicly available due to privacy or ethical restrictions.
